# Exploiting Publicly Available Biological and Biochemical Information for the Discovery of Novel Short Linear Motifs

**DOI:** 10.1371/journal.pone.0022270

**Published:** 2011-07-20

**Authors:** Ahmed Sayadi, Leonardo Briganti, Anna Tramontano, Allegra Via

**Affiliations:** 1 Department of Physics, Sapienza University of Rome, Rome, Italy; 2 Department of Biology, University of Rome “Tor Vergata”, Rome, Italy; 3 Istituto Pasteur Fondazione Cenci Bolognetti, Sapienza University of Rome, Rome, Italy; The Centre for Research and Technology, Hellas, Greece

## Abstract

The function of proteins is often mediated by short linear segments of their amino acid sequence, called Short Linear Motifs or SLiMs, the identification of which can provide important information about a protein function. However, the short length of the motifs and their variable degree of conservation makes their identification hard since it is difficult to correctly estimate the statistical significance of their occurrence. Consequently, only a small fraction of them have been discovered so far. We describe here an approach for the discovery of SLiMs based on their occurrence in evolutionarily unrelated proteins belonging to the same biological, signalling or metabolic pathway and give specific examples of its effectiveness in both rediscovering known motifs and in discovering novel ones. An automatic implementation of the procedure, available for download, allows significant motifs to be identified, automatically annotated with functional, evolutionary and structural information and organized in a database that can be inspected and queried. An instance of the database populated with pre-computed data on seven organisms is accessible through a publicly available server and we believe it constitutes by itself a useful resource for the life sciences (http://www.biocomputing.it/modipath).

## Introduction

Short Linear Motifs (SLiMs) are sub-sequences of few adjacent amino acids (typically between three and ten residues in length) contributing to the molecular function of proteins. SLiMs have been estimated to mediate 15%–40% of protein-protein interactions [1], [2] and recognized to be critical for many biological processes (e.g. sub-cellular targeting, post-translational modification, signal transduction, etc.) [3]. Protein domain-SLiM interactions have also been linked to several diseases, such as Alzheimer [4] and Huntington [5] diseases, Muscular Dystrophy [6], and malaria [7], [8]. Examples of SLiMs are the C-Mannosylation site WxxW [9], the PxxP SH3 domain binding motif [10], [11], the KDEL Golgi-to-Endoplasmic Reticulum retrieving signal [12], the polyproline rich peptides interacting with WW domains [13] and phosphorylation sites [14]. Given their short length, their variable degree of conservation (positions may be degenerate in terms of permitted amino acids), their weak binding affinity [1], the difficulty of correctly estimate the statistical significance of their occurrence in protein sequences, and the fact that most of them reside in disordered regions [15], SLiMs are difficult to discover both experimentally and computationally (e.g. [16]). For this reason, only few hundreds of motifs are known as of today while it is believed that the majority of SLiMs have still to be discovered (e.g. [16]).

Most of the known SLiMs are deposited in manually annotated repositories including PROSITE [17], ELM [18] and MnM [19]. The manual annotation of motifs is an important process that, besides being instrumental as a guide to experimentalists, allows the construction of benchmarking datasets necessary for the assessment of the performance of motif prediction tools. It is however difficult if not impossible to scale the manual process at the level required for handling high throughput data. The cogent need for *de novo* discovery of SLiMs has prompted the development of automatic motif discovery approaches that can be broadly divided into two types: those that use sequence alignments to identify motifs in evolutionarily *related* proteins (e.g. MEME [20]) and those that use over-representation of motifs in evolutionarily *unrelated* proteins sharing a common functional characteristic. For example, DILIMOT [21], which is based on the TEIRESIAS [22] combinatorial pattern discovery algorithm, searches over-represented motifs in non-homologous proteins with a common interaction partner. The MoVIN server [23] is based on the same principle and identifies the presence of common motifs in proteins interacting with the same partner. SLiMDisc [24] uses TEIRESIAS to find shared motifs in all (homologous and non-homologous) proteins with a common attribute (biological function, sub-cellular location, or a common interaction partner); identified common substrings are subsequently weighted according to the evolutionarily relationships of the proteins containing the motif.

SLiMFinder [25] is a combined software package that implements two algorithms, SLiMBuild and SLiMChance. The former is designed to identify motifs that are shared by unrelated proteins whereas the latter calculates a score that accounts for the probability that a given motif occurs in a dataset of unrelated proteins by chance. In practice, the motifs identified by SLiMBuild are returned with a significance value provided by SLiMChance. SliMFinder allows the search to be restricted to specific regions of the set of input proteins such as disordered or non-disordered subsequences, positions annotated by UniProt features and low complexity regions.

The rationale behind most available SLiM discovery systems is the assumption that motifs mediate transient interactions, and therefore play a key role in signalling pathways, the proteins of which often contain (e.g.) SH2, SH3, PTB, 14-3-3 domain interacting motifs. Less well established is whether SLiMs are equally important in mediating interactions in metabolic pathways, which is in principle very likely. In a metabolic pathway a principal chemical is modified by a series of reactions carried out by the proteins of the pathway which therefore interact with either the principal chemical or one of its derivatives. Furthermore, specific reactions in a metabolic pathway are temporally and spatially compartmentalized [26].

It is therefore reasonable to expect that the corresponding proteins and enzymes, or a subset of them, may share a binding motif and/or one or more common cellular localization motifs and that the inspection of the sequence of proteins involved in a common pathway might be very useful for the discovery of novel functional motifs. This is the strategy followed by the procedure described here and we show that it is indeed possible to discover novel motifs shared by proteins involved in the same biological (signalling or metabolic) pathway.

In our procedure, named MoDiPath, proteins are grouped according to the KEGG Pathway Database [27]. The database contains both metabolic pathways (e.g. fatty acid biosynthesis, purine metabolism), based on indirect protein-protein interactions, and non-metabolic pathways (e.g. secretory, signaling pathways), based on direct protein-protein interactions.

MoDiPath identifies over-represented SLiMs in KEGG pathways in different organisms, and uses functional and structural annotation to assess their plausibility. By applying this protocol to seven organisms, we could both re-discover previously known motifs and detect several novel ones. The discovered motifs, annotated with functional, structural and evolutionary conservation information and linked to several other SLiM resources, are stored in a publicly available database accessible through a Web interface (http://www.biocomputing.it/modipath).

The automatic procedure can be downloaded from http://www.biocomputing.it/modipath/MoDiPath.11-04-2011.zip and installed locally.

## Results

### The MoDiPath procedure

The MoDiPath procedure is designed to search for motifs that are over-represented in a set of unrelated proteins belonging to the same biological pathway.

We applied the procedure to all KEGG pathways from seven organisms (*H.sapiens*, *R.norvegicus*, *M.musculus*, *D.melanogaster*, *C.elegan*s, *S.cerevisiae*, *E.coli*) and made these pre-computed data available via a web server.

**Figure 1:**
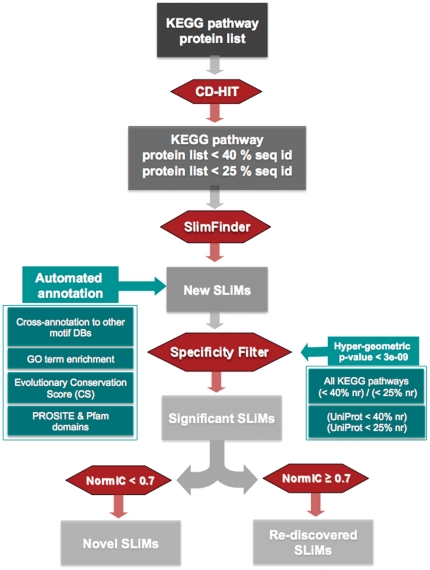
Flowchart of the MoDiPath procedure. NormIC is the CompariMotif [32] similarity score. The CompariMotif tool was used to find similarities between motifs automatically discovered by MoDiPath and motifs already annotated in other databases.

The pipeline consists of the following steps (see Figure 1 and Materials and Methods):

1. Sets of proteins belonging to a given pathway in a given organism are collected (Table S1.1 and S1.2);
2. The proteins are filtered to restrict the analysis to proteins that share not more than 40% and 25% sequence identity and that are therefore less likely to be evolutionarily related (Table S1.1 and S1.2). The 25% threshold was selected since it is commonly used for safe removal of homologous proteins (e.g. [28], [29]). We also allow the user to increase the threshold up to 40%, the lower level of redundancy used, for example, by CD-HIT [30];
3. the SLiMFinder algorithm is used for the identification of over-represented SLiMs shared by all (or a subset of) non-redundant proteins belonging to the pathway;
4. the specificity of the identified motifs is assessed by comparing the number of motif occurrences in the set of proteins belonging to the pathway with that obtained from searching the motifs in the whole set of KEGG protein sequences and in the UniProt knowledge database [31];
5. motifs are ranked based on their hyper-geometric p-value (see later) and pathway-specific ones are identified;
6. motifs are compared with known SLiMs in other databases and annotated with functional, structural and evolutionary conservation information;
7. the annotated motifs are stored in the MoDiPath database.

### Re-discovered and newly discovered motifs

The MoDiPath procedure was able to uncover and re-discover a significant number of motifs (Table 1).

**Table 1:** Number of motifs predicted in KEGG pathways.

Species	Total(a)			Significant SLiMs(b)			Novel SLiMs(c)		
	Total	MP	NMP	Total	MP	NMP	Tot	MP	NMP
***H.sapiens***	2097	836	1261	104	21	83	22	6	16
***M.musculus***	2094	882	1212	127	38	89	28	12	16
***R.norvegicus***	1863	809	1054	72	19	53	15	5	10
***D.melanogaster***	1391	632	759	35	5	30	4	0	4
***C.elegans***	1050	610	440	32	12	20	6	6	0
***E.coli***	933	733	200	11	10	1	2	1	1
***S.cerevisiae***	889	584	305	20	15	5	3	2	1

(a) : Total number of motifs predicted by SliMFinder in KEGG pathways;

(b) : number of significantly over-represented motifs in pathways with respect to the two reference datasets (hyper-geometric p-value<3e-9, see Materials and Methods);

(c) : number of significant motifs that are novel (hyper-geometric p-value<3e-9, NormIC<0.7). MP: Metabolic pathways; NMP: Non-Metabolic Pathways.

We found 104 statistically significant motifs specific to human pathways (21 in metabolic and 83 in non-metabolic pathways). Out of these 104 motifs, 82 have some degree of similarity to already known motifs present in other databases. We define two motifs to be similar if their CompariMotif score [32] is above 0.7 (see Materials and Methods). CompariMotif takes into account exact matches, variants of degenerate motifs and complex overlapping motifs.

Sixty-three of these motifs are identical to known motifs stored in one of the following databases: ELM [18], MnM [19], PhosphoMotif Finder [33], a dataset of motifs extracted from the literature, and a set of SLiMs predicted by Neduva and Russell [1]. Interestingly, twenty-two SLiMs are novel and share no similarity with any known motif. Table 1 shows the number of detected SLiMs already present in existing databases or very similar to one of their entries as well as the number of newly discovered SLiMs in each analysed organism. Novel motifs are reported in Table S2.1 (novel motifs detected in the 25% non-redundant dataset of sequences) and Table S2.2 (novel motifs detected in the 40% non-redundant dataset of sequences) and re-discovered motifs are reported in Table S3.1 (known motifs detected in the 25% non-redundant dataset of sequences) and Table S3.2 (known motifs detected in the 40% non-redundant dataset of sequences).

Table 2 reports the total number of KEGG pathways analysed per species and the number of pathways for which at least one SLiM has been detected.

**Table 2:** Number of KEGG pathways (total and with motifs).

	KEGG pathways(a)			Pathways with SLiMs(b)			Pathways with novel SLiMs(c)		
Species	Total	MP	NMP	Total	MP	NMP	Total	MP	NMP
*H.sapiens*	201	87	114	42	13	29	19	5	14
*M.musculus*	198	87	111	50	17	33	18	7	11
*R.norvegicus*	197	84	113	38	13	25	14	5	9
*D.melanogaster*	118	84	34	9	4	5	3	0	3
*C.elegans*	117	82	35	15	9	6	4	4	0
*E.coli*	105	90	15	8	7	1	2	1	1
*S.cerevisiae*	92	70	22	11	9	2	2	1	1

(a) : Total number of KEGG pathways in each of the seven organisms under study;

(b) : Number of KEGG pathways for which at least one significant motif was found (hyper-geometric p-value<3e-9, see Materials and Methods);

(c) : Number of KEGG pathways for which at least one statistically significant novel motif was found (i.e. a motif with no similarity to any known motif) (hyper-geometric p-value<3e-9, NormIC<0.7). MP: Metabolic pathways; NMP: Non-Metabolic Pathways.

Motifs were also compared to each other (all-against-all) in order to group similar motifs identified by CompariMotif (CompariMotif score ≥0.7). The data reported in Table 1 were filtered by taking into account only one representative motif (motif *representative*) for each similarity group and the results are shown in Table 3, from which it can be appreciated that there are 64 statistically significant motifs specific for human pathways (18 in metabolic and 46 in non-metabolic pathways). More detailed information obtained from the all-against-all motif comparison is reported in Tables S2.1 and S2.2 (for novel motifs) and Tables S3.1 and S3.2 (for known motifs).

**Table 3:** Number of motif representatives predicted in KEGG pathways.

Species	Total(a)			Significant SLiMs(b)			Novel SLiMs(c)		
	Total	MP	NMP	Total	MP	NMP	Tot	MP	NMP
***H.sapiens***	813	329	484	64	18	46	21	6	15
***M.musculus***	803	384	419	58	20	38	22	10	12
***R.norvegicus***	727	322	405	55	16	39	15	5	10
***D.melanogaster***	616	378	238	14	5	9	4	0	4
***C.elegans***	513	307	206	20	11	9	5	5	0
***E.coli***	465	378	87	7	6	1	2	1	1
***S.cerevisiae***	502	336	166	16	13	3	2	1	1

(a) : Total number of motif *representatives* predicted by SliMFinder in KEGG pathways;

(b) : number of significantly over-represented motif *representatives* in pathways with respect to the two reference datasets (hyper-geometric p-value<3e-9, see Materials and Methods);

(c) : number of significant motif *representatives* that are novel (hyper-geometric p-value<3e-9, NormIC<0.7). MP: Metabolic pathways; NMP: Non-Metabolic Pathways.

Data reported in Tables 1, 2, and 3 refer to the 40% non-redundant sequence dataset. The corresponding data for the 25% non-redundant dataset can be found in the Supporting Information S1 file.

### Evolutionary conservation of SLiMs

Evolutionary conservation is often used for assessing the biological significance of predicted SLiMs. It is reasonable to expect that if the residues composing a motif have a functional role, the motif is evolutionary conserved. On the other hand, SLiMs are usually short, tend to localise in disordered regions that are difficult to align, and might not be shared even by closely related sequences as a result of single mutations. These observations imply that it is difficult to trace their evolutionary history. Here, we use a scoring scheme that has been specifically designed for SLiMs [34] taking into account the potential problems mentioned above. We also used the CompariMotif algorithm to highlight motifs that are shared by two or more of the species under study (Table S4.1 and Table S4.2). We found that, with some exceptions, motifs shared by different organisms are related to similar or identical pathways. Fifty-five (45 known and 10 novel) out of the 104 human specific motifs are shared by proteins belonging to the same pathway in at least another species in the 40% sequence dataset (Table S4.2).

### Assessment of some re-discovered and newly discovered motifs

We manually analysed a number of examples extracted from the list of re-discovered SLiMs (Table S3.1 or Table S3.2) detected by the MoDiPath procedure to verify the effectiveness of our procedure.

Several of the automatically identified motifs listed in Table S4.1 or S4.2 (SLiMs shared by two or more than two species under study) are variations of the SKL$ theme, where S represents a serine, K a lysine, L a leucine, and $ indicates that true positive occurrences of the motif are found at the carboxy-terminal of proteins.

The SKL$ motif significantly overlaps with the ELM TRG_PTS1 motif (regular expression: (.[SAPTC][KRH][LMFI]$)), which is annotated as a C-terminal signal interacting with the Pex5p protein to target proteins into the peroxisomal matrix, and is identical to a MnM motif annotated as Pex5 binding and associated to trafficking to Peroxisomes. Furthermore, Gould et al [35] identified the motif as a peroxisomal targeting signal in four unrelated peroxisomal proteins and both Miura et al [36] and Fujiki [37] found, more generally, that it functions as a topogenic signal in the translocation of proteins into peroxisomes. The signal needs to include the whole tripeptide sequence with a free alpha-COOH group at its carboxy terminus.

This motif is significantly over-represented in the Peroxisome KEGG pathway (KEGG ID: hsa04146) and specific (hyper-geometric p-value<1.72e-11). Six proteins out of the sixty-nine belonging to this pathway share the motif. All of them are localized in the peroxisome, five of them participate to a fatty acid metabolic process and three of them have catalytic activity. Figure S1 shows the PROSITE [17] and Pfam [38] domain composition of these proteins together with the position of the SKL$ motif in the sequence. Notably, the motif occurs in only 8 other sequences out of the 14,239 proteins of the non-redundant UniProt human dataset (filtered at the 40% sequence identity level). Of these, four are membrane or secreted proteins and therefore are likely to be false positives. The remaining four proteins are a peroxisomal acyl-coenzyme A oxidase 3 (UniProt O15254-1), a Lon protease homolog (Q86WA8), a peroxisomal leader peptide-processing protease (Q2T9J0), and a zinc-binding alcohol dehydrogenase domain-containing protein (Q8N4Q0). O15254-1 is a different isoform of O15254-2, a human protein, reported to belong to the hsa04146 KEGG pathway, that does not contain the motif and differs from O15254-1 for the lack of the last 75 C-term amino acids; it is not clear why O15254-2 was chosen for inclusion in the KEGG hsa04146 pathway; we argue that O15254-1 should be added to the KEGG hsa04146 pathway and the assignment of O15254-2 reassessed. Q86WA8 is annotated in UniProt for having the SKL$ targeting motif and its cellular compartment is known to be the Peroxisome, but is not associated with any KEGG pathway. Q2T9J0 and Q8N4Q0 are peroxisomal proteins but they are neither annotated for having the motif nor associated with any KEGG pathway. We propose that Q2T9J0 and Q8N4Q0 use the SKL$ motif as targeting signal to the peroxisome and suggest that their inclusion, and that of Q86WA8, in the KEGG peroxisome pathway should be considered.

**Figure 2:**
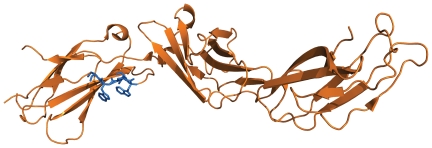
The crystal structure of the human granulocyte colony-stimulating factor (GCSF) receptor. The structure of the GCSF receptor (PDB:2D9Q [42]) is reported in orange. Residues corresponding to the WS.WS motif (residues 295–299) are shown in blue.

Another interesting motif that we automatically detected is WS.WS (Trp-Ser-any-Trp-Ser), which is specific for the Hematopoietic cell lineage pathway (KEGG ID: hsa04640) (hyper-geometric p-value<3.10e-11). The motif was found in the analysis of both the 40% and 25% non-redundant sequence datasets and is present in 9 proteins out of the 79 belonging to the pathway, whereas it occurs in only 59 other sequences of the 40% non-redundant UniProt human dataset. Figure S2 shows the PROSITE [17] and Pfam [38] domain composition of the nine KEGG proteins together with the position of the WS.WS motif in the sequence: the motif is found at the C-terminal of the PROSITE FN3 domain in six cases and outside of the domain in three cases. This suggests that, at least in some of these proteins, the occurrence of the motif is not due to evolutionary conservation but rather to functional contraints. The WS.WS motif appears to be necessary for the binding activity of the erythropoietin receptor (EpoR), a member of the cytokine and growth factor receptor family. These proteins share conserved features in their extracellular and cytoplasmic domains presumably necessary for proper folding and thereby efficient intracellular transport and cell-surface receptor binding. Yoshimura et al [39] demonstrated that mutations in the motif of EpoR abolish processing, ligand binding, and activation of the receptor, while Schimmenti et al [40] showed that WS.WS is necessary for EpoR binding to Epo. For two (Uniprot: P15509 and Q99062) out of the nine proteins hosting the motif, the crystal structure has been determined (PDB:3CXE [41] and 2D9Q [42]). In both cases, the motif instance is nicely found in an exposed loop of the protein structure (Figure 2).

The proteins belonging to the hematopoietic cell lineage pathway (KEGG ID: hsa04640) and sharing the motif all take part in two other pathways: Cytokin-cytokine receptor interaction (KEGG: hsa04060) and Jak-STAT signaling pathway (KEGG: hsa04630).

Our analysis also revealed that, out of the 59 other sequences of the non-redundant UniProt human dataset having the motif, 32 are likely to be false positives. The eighteen remaining proteins, that we estimated to be false negatives, have a similar molecular function (receptor activity) and a similar subcellular localization (membrane or secreted) of the true positives. Moreover, 16 of them are annotated in Uniprot as having the functional motif, 13 are involved in both hsa04060 and hsa04630 KEGG pathways, one (Q14627) belongs to hsa04630, three (O75462, Q8IUI8, Q8NI17) are included in KEGG but without pathway annotation, one (P40189) is not present in KEGG.

From the examples reported above and others reported in Tables S3.1 and S3.2, it is apparent that our automatic analysis can effectively discover biologically significant motifs and therefore that some of the novel ones (Tables S2.1 and S2.2), i.e. motifs not annotated in any other resource, might be interesting and worth investigating.

No matter how stringent are the statistical parameters used to identify significant hits, assessing the biological value of a short motif can only be achieved via experimental validation or by a carefully reviewing of the literature.

As an example of the usefulness of inspecting our proposed novel motifs and of the procedure that one can follow to gain confidence in the results, we illustrate here the case of the [FL].L.C..Y..A motif. This is conserved both in human (hsa04666) and mouse (mmu04666) Fc gamma R-mediated phagocytosis KEGG pathways. In the following we discuss the analysis of the human proteins sharing the motif, but the results are the same for the mouse proteins (data not shown).

The motif is present in 5/63 human proteins belonging to hsa04666: P42338, Q9Y217, Q13393, Q92608, O14939. P42338 is the catalytic subunit beta isoform of the phosphatidylinositol-4,5-bisphosphate 3-kinase, which phosphorylates several phosphoinositides [phosphatidylinositol (PtdIns), phosphatidylinositol 4-phosphate (PtdIns4P), phosphatidylinositol 4,5-bisphospate (PtdIns(4,5)P2)] with a preference for PtdIns(4,5)P2. Phosphoinositides represent a small fraction of cellular phospholipids and are very important regulatory molecules utilized both as cellular membrane structural lipids and as precursors of multiple signalling molecules. Q9Y217 is a 1-phosphatidylinositol-3-phosphate 5 kinase. Q13393 and O14939 are phospholipases, which UniProt reports to be stimulated by PtdIns(4,5)P2 and PtdIns(3,4,5)P3 and by PtdIns(4,5)P2, respectively. Q92608 is a Dedicator of cytokinesis protein 2 (DOCK2). Interestingly, Nishikimi and colleagues [43] found that DOCK2 rapidly translocates to the plasma membrane in a PtdIns(3,4,5)-P3 dependent manner. In summary, all these proteins are involved in the interaction with phosphoinositides. By searching the motif in the whole set of human UniProt sequence, we found 9 additional occurrences in 9 different proteins. Three of them are isoforms of Q13393 and two are isoforms of O14939. Of the remaining four, one (O00329) is a PtdIns(4,5)P2 3-kinase catalytic subunit delta isoform, which is reported to be involved in the PtdIns phosphate biosynthesis, and one (Q8TDW7) is the Protocadherin FAT-3. The molecular function of FAT-3 is not well known, however some authors [44], [45] reported that the fat-3 gene acts in the same genetic pathway as synaptojanin, the main substrate of which in the brain is PtdIns(4,5)P2 and suggest that FAT-3 functions in the endocytic part of the synaptic vescicles recycling process. More specifically, Marza et al [45] found that the levels of PtdIns(4,5)P2 at release sites are increased in *Caenorhabditis elegans* fat-3 mutants lacking long-chain polyunsaturated fatty acids (LC-PUFAs), which would suggest that fat-3 influences the levels of PtdIns(4,5)P2 at release sites. For the remaining two proteins (O75976 and Q8NEZ3) we did not find any clue to deduce potential interactions with phosphoinositides and we cannot exclude that they are false positives. We also analysed the 58/63 hsa04666 proteins that do not have the [FL].L.C..Y..A motif. In this case, we automatically selected proteins that have at least one keyword related to phosphoinositides (e.g. PtdIns) in their UniProt annotation: we found ten of such proteins and inspected their sequences. In six of them, we found motifs that are similar, although not identical, to [FL].L.C..Y..A. For example, the P48736 sequence contains the subsequence FVYSCAGYCVA which could be described by the [FL].[LY].C..Y..A regular expression, a less specific version of the original expression. In the four remaining sequences, we did not find sub-sequences sufficiently similar to the identified motif.

In conclusion, our analysis suggests that the [FL].L.C..Y..A motif (and perhaps other related ones) is involved or participates in the recognition of phosphoinositides.

### The MoDiPath Database and the Web Interface

The whole set of motifs identified by our procedure in the seven analysed organisms is stored in a MySQL database and made available to the scientific community through a Web Interface (http://www.biocomputing.it/modipath). Data are available for motifs identified in both the 40% and 25% datasets. The Web Interface has two main sections: “Search”, for searching the MoDiPath database, and ‘Scan’, for either searching motif matches in a protein sequence submitted by the user or for scanning the database with a user-defined regular expression. The MoDiPath database can be searched by KEGG pathway identifier, protein identifier (either UniProt or KEGG) and/or organism. The search by KEGG ID returns a table reporting the motif(s) associated with the input pathway. For each motif, the output provides the motif regular expression, indicates if the regular expression overlaps with at least one motif in another database (ELM, MnM, etc), reports the hyper-geometric p-value of the motif with respect to the SwissProt dataset (see Materials and Methods) and the fraction of proteins belonging to the pathway that contain the motif.

The system also provides further information on a specific motif, including

- the SlimFinder motif statistics;
- the sequence alignment and the list of proteins that both belong to the pathway AND contain the motif;
- the motif cross-reference to other databases of motifs;
- the list of GO terms shared by the proteins matching the motif;
- PROSITE [17] and Pfam [38] domains shared by the protein sequences matching the motif;
- the exact sequence of the motif;
- the starting and ending position of the match in the protein sequence;
- the evolutionary conservation score;
- the PDB ID (if available).
- access to the STRING database [46] that provides an interaction map specific for the proteins of the pathway sharing the motif.

**Figure 3:**
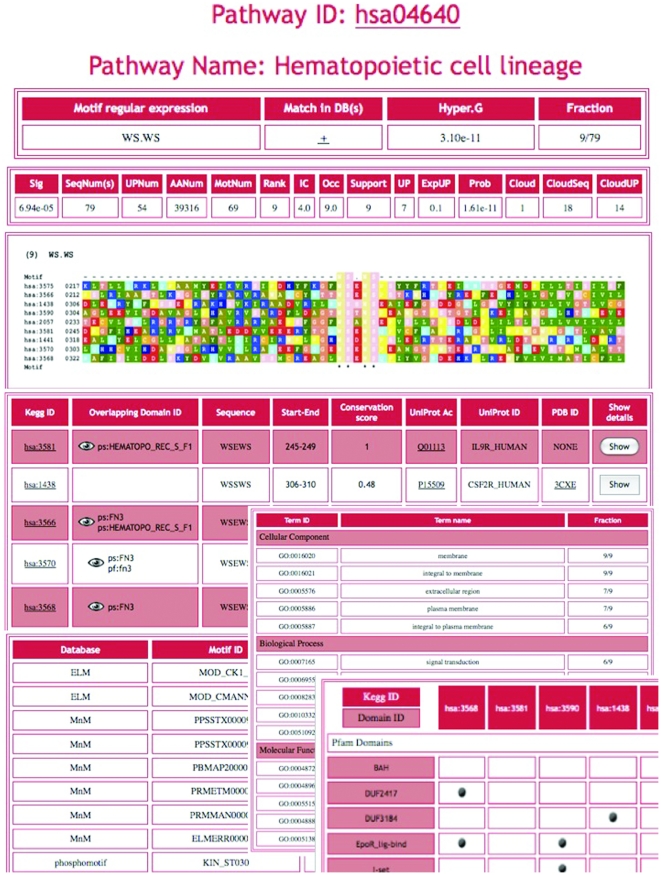
The information provided by MoDiPath for the hsa04640 KEGG pathway. (a) First column: the SLiM regular expression; Second column: a ‘+’ is reported if the motif overlaps to a similar motif in other databases (the list of which is shown by moving the mouse over the ‘+’); Third column: the hyper-geometric p-value of the number of motif hits in the hsa04640 pathway compared to the number of motif hits in the SwissProt database; Fourth column: The fraction of proteins in the hsa04640 pathway that contain the WS.WS motif (b) Multiple sequence alignment of the hsa04640 pathway proteins containing the WS.WS motif. (c) Information about each of the hsa04640 proteins containing the WS.WS motif. Clicking on the ‘Show’ button provides more detailed information, including the protein structure visualization with the motif hit(s) highlighted. (d) List of motif overlap(s) to similar motifs in other databases; the last column reports the CompariMotif [32] similarity score (NormIC). (e) GO terms shared by the hsa04640 pathway proteins that have the motif; the last column reports the fraction of the proteins hosting the motif that share a GO term.

Figure 3 shows a screenshot with the information provided by MoDiPath for the WS.WS motif, which is specific for the hsa04640 KEGG pathway. For each protein sharing the motif, a page containing functional and structural details is provided. In particular, if the protein is of known structure, the position of the matching sub-sequence is displayed in the context of its three-dimensional structure.

If the initial search is performed with a protein ID, the list of pathways including the query protein and, for each pathway, the list of motifs matching the protein, if any, can be retrieved.

A search by organism returns the list of KEGG pathways for which at least one statistically significant motif has been found in the query organism. Each pathway is linked to the complete list of its motifs.

Finally, for each motif it is possible to download, explore and edit the whole pathway map corresponding to a selected motif using KGML-ED [47], a Web Java start program downloadable through the MoDiPath Web Interface. In each pathway map, proteins containing the motif are conveniently highlighted.

The implementation of the complete system can also be downloaded and installed locally to analyse other organisms of interest or to use definition of pathways provided by other resources such as PANTHER [48], REACTOME [49], or EcoCyc and MetaCyc databases [50].

## Discussion

The discovery of linear motifs is a difficult task that usually requires the identification of a set of non-homologous proteins sharing a common functional feature (e.g., an interaction partner or a cellular compartment). Many algorithms for motif discovery are nowadays available and appropriate statistics have been developed for estimating the effectiveness of a motif for function prediction. However, several challenging aspects still remain, for example one needs to identify appropriate sets of non-homologous proteins sharing a functional feature and associate the appropriate biological function to newly discovered motifs. The two issues are of course strictly related: for example, if one were able to identify a set of proteins that are targeted to the same cellular compartment, a motif significantly over represented in their sequences would be likely to be a targeting signal to that compartment.

This is the idea that inspired several works in the field, such the one of Neduva et al, aimed at discovering motifs that mediate protein-protein interaction networks [51].

Restricting the analysis to non-homologous proteins is relevant to avoid detecting general sequence homology features instead of genuine functional motifs. Even though functional motifs can also be found in evolutionary related proteins, the most interesting ones are represented by cases of convergent evolution. However, the latter are rare and difficult to discover, especially at the level of the protein sequence. One possible approach to idenitfy motifs arising independently during evolution, consists, on one hand, in using non-homologous sequences and, on the other, in filtering out motifs occurring in similar (e.g. Pfam) domains. The MoDiPath database only collects motifs identified in non-redundant sets of proteins and annotates motif matching proteins for the presence of Pfam and PROSITE domains. This facility does not ensure that every discovered motif will be a case of convergent evolution, but can help users identify those that are likely to be relicts of common descent with no specific functional properties.

Here, we focused on functional features typical of metabolic and signaling pathways. Pathway functional features can be of different types: they could be related to the interaction with the same metabolite or its derivatives, or pertain to specific cellular compartments, or arise, for example, from the interaction with recurring signaling modular domains (SH2, SH3, WW, PDZ, etc).

We used this strategy to explore all proteins of seven organisms assigned to KEGG pathways and identified a number of potentially biologically significant motifs that represent a valid starting point for further computational and experimental functional investigation.

The methodology is reliable, as demonstrated by the fact that we can automatically re-discover known motifs, for example the targeting peroxisome signal SLK$ or the WS.WS motif necessary for processing, ligand binding and activation of receptors specific for the hematopoietic cell lineage pathway but also taking part in two related pathways: the cytokin-cytokine receptor interaction pathway and the Jak-STAT signaling pathway.

The procedure is also effective in detecting novel motifs. As an example we described here the analysis of one of them ([FL].L.C..Y..A) for which no functional annotation is available, and found that it is likely to be involved in the recognition of phosphoinositides.

We hope that MoDiPath, its associated database as well as the list of motifs that we provide here will contribute to speed up the discovery of novel motifs and will constitute a useful resource for the life scientists.

## Materials and Methods

### Motif discovery procedure

We used the KEGG (Kyoto Encyclopedia of Genes and Genomes) Pathway database as the source of pathway information. In this resource, proteins from 1173 different species (release of March 2010) [27] are clustered in pathways. Each pathway represents functional aspects of a biological system, and involves a specific protein list, graphically represented as a network of connected proteins. The number of pathways depends on the species (Table 2).

Pre-computed data presently associated with MoDiPath are available for seven species (*H.sapiens*, *R.norvegicus*, *M.musculus*, *D.melanogaster*, *C.elegan*s, *S.cerevisiae*, *E.coli*).

For each KEGG pathway, we collected all protein sequences and, in order to only retain unrelated proteins, used CD-HIT (last release 4.0 beta) [30] to remove redundancy at the 40% as well as at the 25% identity level. Each pathway protein list was analysed by SlimFinder, one of the best performing tools for linear motif detection [25]. In SliMFinder the term SLiM is used to mean short (generally less than 10 residues), linear (i.e. made up of adjacent residues in the primary sequence) true functional motif. SLiMs, which are encoded by regular expressions, are composed by defined amino acid positions often separated by wildcards (which represent positions that can be occupied by any amino acid). Defined position can be fixed (only one amino acid type is permitted) or degenerate (more than one amino acid type is permitted). The number of defined positions and of wildcards can be either fixed or variable.

SliMFinder is a software package that implements two different algorithms, SliMBuild and SliMChance, and offers a number of input masking options, which can be used to restrict the analysis to specific parts of the proteins, such as disordered or low complexity regions. SliMBuild builds motifs by first combining pairs of residues into longer patterns and subsequently incorporating amino acid degeneracy and/or variable length wildcards, until the SLiM matches the desired number of unrelated sequences. SliMChance deals with the probability that a motif occurs in a sequence dataset by chance and determines a score indicating how unlikely a given motif is compared to other motifs in a dataset.

The input of SLiMFinder is a user-defined set of sequences, plus a number of options such as the BLAST e-value threshold to be used to identify which input proteins are related to which other input proteins, the minimum number of unrelated proteins that should contain the motifs, the maximum number of defined positions in a motif, the maximum number of wildcard positions, disorder masking, etc.

SLiMFinder was run locally with default parameters except for the disorder masking option, which was deactivated. We retained the subset of top significant motifs with a very high probability of significance (SLiMChance probcut = 0.99).

The statistical assessment of a motif specificity for a given pathway was obtained by comparing the number of the motif occurrences in the proteins belonging to the pathway with the number of occurrences in two reference datasets: 1) all UniProt proteins (from the same organism) and 2) all the proteins included in KEGG. Since proteins belonging to a KEGG pathway are contained in both reference datasets, the hyper-geometric p-value was used to assess the motif specificity, i.e. to assess whether it is observed more frequently in the KEGG pathway than expected by chance given its frequency in each of the two reference datasets.

**Figure 4:**
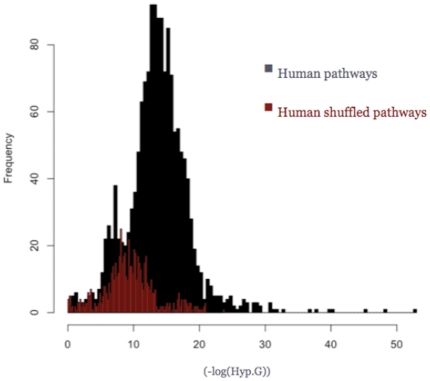
Motif occurrence Hyper-geometric distribution. Hyper-geometric p-value distribution for the number of motif occurrences in true (black) and reshuffled (red) KEGG pathways with respect to the number of motif occurrences in the UniProt dataset for *H.sapiens*. The p-value = 3e-9 approximately corresponds to a false discovery rate of 10%.

In order to choose an un-biased hyper-geometric p-value threshold, we had to take into account the KEGG pathway peculiar composition, which is clearly not random. To this aim, we built random pathways by reshuffling the proteins of each pathway with proteins belonging to other pathways, leaving the number of proteins per pathway unmodified. Next, we plotted the hyper-geometric p-value distribution of motif occurrences in the random datasets with respect to their occurrences in the Uniprot dataset and compared it to the corresponding distribution for the true datasets (Figure 4). We estimated that the hyper-geometric p-value that better discriminates between true and false positives (random) is 3e-9, which corresponds to a false discovery rate (FDR) lower than 10%. The procedure was repeated ten times for *H.sapiens* producing essentially the same result. The result was the same when all human proteins of SwissProt were used for reshuffling (data not shown).

### Motif-motif comparison

The CompariMotif software [32] was used to compare predicted motifs to similar motifs annotated in other databases (ELM [18]), MnM [19], PhosphoMotif Finder [33]), a set of SLiMs extracted from the literature, and predicted SLiMs from Neduva & Russell [1]). The software takes as input two lists of motifs and returns a set of motif pairs associated with a similarity score (Normal IC), which ranges between 0.0 (weak similarity) and 1.0 (strong similarity).

CompariMotif uses a sliding window to compare every possible alignment between two motifs (represented as regular expressions). Two aligned positions are considered a mismatch if they have no amino acid in common amino (in which case the motif pair is rejected). Each compared position is scored according to its information content: IC_i_ = −log_N_(f_a_), where IC_i_ is the information content for position *i*, f_a_ is the summed frequency for the amino acids at position *i*, and N = 20. IC_i_ is a modification of the Shannon's Information Content algorithm [52] where wildcards have score 0, fixed positions have score 1, and ambiguous positions have scores between 0 and 1. The IC_m_ of a match is the sum of the component IC_i_ values. A sliding window will produce several matches and the best match is taken as the one with the best overall IC_m_. In order to make the score independent from the length and degeneracy of the matching motifs, a final normalized IC (Norm IC) score is calculated by dividing the IC_m_ by the lower IC value for the two motifs. Pairs of motifs with Norm IC = 0 are clearly dissimilar and pairs of motifs with Norm IC = 1 are highly similar, however, a cut-off must be set for pairs of motifs with intermediate Norm IC values in order to discriminate between true and false matches. The choice of such cut-off is arbitrary and depends on the empirical observation of compared motifs (RJ Edwards, personal communication).

Based on the analysis of Norm IC scores for MoDiPath pairs of compared motifs, we considered two SLiMs to be similar if their Normal IC >0.7.

## Supporting Information

Supporting Information S1 **Supplementary motif and pathway statistics.** The file contains the same data of Table 1, 2, and 3 (main text) calculated for the 25% non-redundant sequence dataset. Moreover, it reports statistics on motifs occurring in disorered and loop regions. It is organized in three sections as follows: 1) Motif and pathway statistics calculated for the 25% non-redundant sequence dataset. 2) Statistics of motifs occurring in disordered regions of proteins (calculated for both the 40% and 25% datasets). 3) Statistics on motifs occurring in loop regions (calculated for both the 40% and 25% datasets). A motif is assigned to a loop (disordered) region if at least 50% of the residues belonging to the motif true positive matches are in loop (disordered) regions, respectively.
(DOC)

Table S1 **Total number of proteins belonging to the pathways under study and number of motifs per pathway.** Table S1.1: Data obtained from the analysis of the 25% non-redundant sequence dataset. Table S1.2: Data obtained from the analysis of the 40% non-redundant sequence dataset.
(XLS)

Table S2 **List of novel motifs.** Table S2.1: List of novel motifs obtained by restricting the analysis to the 25% non-redundant sequence dataset. Table S2.2 – List of novel motifs obtained by restricting the analysis to the 40% non-redundant sequence dataset.
(XLS)

Table S3 **List of known motifs.** Table S3.1: List of known motifs obtained by restricting the analysis to the 25% non-redundant sequence dataset. Table S3.2: List of known motifs obtained by restricting the analysis to the 40% non-redundant sequence dataset.
(XLS)

Table S4 **List of motifs shared by two or more of the species under study.** Table S4.1: List of motifs shared by two or more of the species under study in the 25% sequence non-redundant dataset. Table S4.2: List of motifs shared by two or more of the species under study in the 40% non-redundant sequence dataset.
(XLS)

Figure S1 **PROSITE and Pfam domain composition of hsa04146 KEGG pathway sequences matching the SKL$ motif.** PROSITE and Pfam domain composition in the (a) 25% and (b) 40% non-redundant sequences belonging to the hsa04146 KEGG pathway and matching the SKL$ motif. Red bars indicate the position of the SKL$ motif in the sequence. Notice that there are no differences between (a) and (b).
(PDF)

Figure S2 **PROSITE and Pfam domain composition of hsa04640 KEGG pathway sequences matching the WS.WS motif.** PROSITE and Pfam domain composition in (a) 25% and (b) 40% non-redundant sequences belonging to the hsa04640 KEGG pathway and matching the WS.WS motif. Red bars indicate the position of the WS.WS motif in the sequence.
(PDF)
