# Supplementary material for: Exploiting Publicly Available Biological and Biochemical Information for the Discovery of Novel Short Linear Motifs
Source: PLoS One. 2011 Jul 20;6(7):e22270. doi: 10.1371/journal.pone.0022270 (PMC3140502; doi:10.1371/journal.pone.0022270)
Supplement: Supporting Information S1 — Supplementary motif and pathway statistics. The file contains the same data of Table 1, 2, and 3 (main text) calculated for the 25% non-redundant sequence dataset. Moreover, it reports statistics on motifs occurring in disorered and loop regions. It is organized in three sections as follows: 1) Motif and pathway statistics calculated for the 25% non-redundant sequence dataset. 2) Statistics of motifs occurring in disordered regions of proteins (calculated for both the 40% and 25% datasets). 3) Statistics on motifs occurring in loop regions (calculated for both the 40% and 25% datasets). A motif is assigned to a loop (disordered) region if at least 50% of the residues belonging to the motif true positive matches are in loop (disordered) regions, respectively. (DOC) [file pone.0022270.s001.doc]

## 1) Motif and pathway statistics calculated for the 25% non-redundant sequence dataset (Tables 1a, 2a and 3a correspond to Table 1, 2, and 3 of the main manuscript, respectively)

**Table 1a** – **Number of motifs predicted in KEGG pathways for the 25% non-redundant sequence dataset**

| **Species** | **Total^(a)^** | | | **Significant SLiMs^(b)^** | | | **Novel SLiMs^(c)^** | | |
| --- | --- | --- | --- | --- | --- | --- | --- | --- | --- |
|  | **Total** | **MP** | **NMP** | **Total** | **MP** | **NMP** | **Tot** | **MP** | **NMP** |
| H.sapiens | 1786 | 750 | 1036 | 86 | 27 | 59 | 12 | 6 | 6 |
| ***M.musculus*** | 1812 | 755 | 1057 | 98 | 20 | 78 | 16 | 9 | 7 |
| ***R.norvegicus*** | 1735 | 726 | 1009 | 52 | 12 | 40 | 10 | 3 | 7 |
| ***D.melanogaster*** | 1241 | 571 | 670 | 26 | 4 | 22 | 4 | 1 | 3 |
| ***C.elegans*** | 881 | 515 | 366 | 35 | 11 | 24 | 3 | 3 | 0 |
| ***E.coli*** | 906 | 705 | 201 | 15 | 12 | 3 | 1 | 0 | 1 |
| ***S.cerevisiae*** | 800 | 531 | 269 | 19 | 17 | 2 | 5 | 5 | 0 |

**(a)**: Total number of motifs predicted by SliMFinder in KEGG pathways; **(b)**: number of significantly over-represented motifs in pathways with respect to the two reference datasets (Hypergeometric p-value < 3e-9, see Materials and Methods); **(c)**: number of significant motifs that are novel (Hyper-geometric p-value < 3e-9, NormIC < 0.7). MP: Metabolic pathways; NMP: Non-Metabolic Pathways.

**Table 2a** - **Number of KEGG pathways (total and with motifs) calculated for the 25% non-redundant sequence dataset**

|  | KEGG pathways^(a)^ | | | **Pathways with SLiMs^(b)^** | | | Pathways with novel SLiMs^(c)^ | | |
| --- | --- | --- | --- | --- | --- | --- | --- | --- | --- |
| **Species** | **Total** | MP | **NMP** | **Total** | **MP** | **NMP** | **Total** | **MP** | **NMP** |
| H.sapiens | 201 | 87 | 114 | 38 | 13 | 25 | 9 | 5 | 4 |
| *M.musculus* | 198 | 87 | 111 | 36 | 15 | 21 | 12 | 7 | 5 |
| *R.norvegicus* | 197 | 84 | 113 | 24 | 9 | 15 | 9 | 3 | 6 |
| *D.melanogaster* | 118 | 84 | 34 | 7 | 3 | 4 | 4 | 1 | 3 |
| *C.elegans* | 117 | 82 | 35 | 10 | 7 | 3 | 2 | 2 | 0 |
| *E.coli* | 105 | 90 | 15 | 11 | 9 | 2 | 1 | 0 | 1 |
| *S.cerevisiae* | 92 | 70 | 22 | 12 | 10 | 2 | 4 | 4 | 0 |

**(a)**: Total number of KEGG pathways in each of the seven organisms under study; **(b)**: Number of KEGG pathways for which at least one significant motif was found (Hyper-geometric p-value < 3e-9, see Materials and Methods); **(c)**: Number of KEGG pathways for which at least one statistically significant novel motif was found (i.e. a motif with no similarity to any known motif) (Hyper-geometric p-value < 3e-9, NormIC < 0.7). MP: Metabolic pathways; NMP: Non-Metabolic Pathways.

**Table 3a - Number of motif representatives predicted in KEGG pathways calculated for the 25% non-redundant sequence dataset**

| **Species** | **Total^(a)^** | | | **Significant SLiMs^(b)^** | | | **Novel SLiMs^(c)^** | | |
| --- | --- | --- | --- | --- | --- | --- | --- | --- | --- |
|  | **Total** | **MP** | **NMP** | **Total** | **MP** | **NMP** | **Tot** | **MP** | **NMP** |
| H.sapiens | 726 | 307 | 419 | 52 | 20 | 32 | 10 | 6 | 4 |
| ***M.musculus*** | 765 | 362 | 403 | 48 | 18 | 30 | 15 | 9 | 6 |
| ***R.norvegicus*** | 755 | 331 | 424 | 33 | 11 | 22 | 10 | 3 | 7 |
| ***D.melanogaster*** | 558 | 346 | 212 | 11 | 4 | 7 | 4 | 1 | 3 |
| ***C.elegans*** | 474 | 304 | 170 | 17 | 9 | 8 | 2 | 2 | 0 |
| ***E.coli*** | 452 | 365 | 87 | 12 | 9 | 3 | 1 | 0 | 1 |
| ***S.cerevisiae*** | 460 | 318 | 142 | 17 | 15 | 2 | 4 | 4 | 0 |

**(a)**: Total number of motif *representatives* predicted by SliMFinder in KEGG pathways; **(b)**: number of significantly over-represented motif *representatives* in pathways with respect to the two reference datasets (Hypergeometric p-value < 3e-9, see Materials and Methods); **(c)**: number of significant motif *representatives* that are novel (Hyper-geometric p-value < 3e-9, NormIC < 0.7). MP: Metabolic pathways; NMP: Non-Metabolic Pathways.

# 2) Statistics on motifs occurring in disordered regions

**Table 4a** **- Number of motifs predicted to be disordered accordingly to IUPred in the 25% non-redundant sequence dataset**

| **IUPred** | **All** | **Significant SLiMs^(b)^** | **Known SLiMs^(c)^** | **Novel SLiMs^(c)^** |
| --- | --- | --- | --- | --- |
| H.sapiens | 115/1786 | 5/86 | 5/74 | 0/12 |
| *M.musculus* | 170/1812 | 40/98 | 38/82 | 2/16 |
| *R.norvegicus* | 119/1735 | 14/52 | 14/42 | 0/10 |
| *D.melanogaster* | 374/1241 | 10/26 | 9/22 | 1/4 |
| *C.elegans* | 119/881 | 22/35 | 22/32 | 0/3 |
| *E.coli* | 3/906 | 0/15 | 0/14 | 0/1 |
| *S.cerevisiae* | 44/800 | 1/19 | 1/14 | 0/5 |

A motif is assigned to a disordered region if at least 50% of the residues belonging to the motif true positive matches are in disordered regions.

Table 4b - Number of motifs predicted to be disordered accordingly to IUPred in the 40% non-redundant sequence dataset

| **IUPred** | **All** | **Significant SLiMs^(b)^** | **Known SLiMs^(c)^** | **Novel SLiMs^(c)^** |
| --- | --- | --- | --- | --- |
| H.sapiens | 164/2097 | 19/104 | 19/82 | 0/22 |
| *M.musculus* | 113/2094 | 9/127 | 8/99 | 1/28 |
| *R.norvegicus* | 127/1863 | 11/72 | 11/57 | 0/15 |
| *D.melanogaster* | 410/1391 | 16/35 | 14/31 | 2/4 |
| *C.elegans* | 146/1050 | 10/32 | 10/26 | 0/6 |
| *E.coli* | 2/933 | 0/11 | 0/9 | 0/2 |
| *S.cerevisiae* | 50/889 | 0/20 | 0/17 | 0/3 |

A motif is assigned to a disordered region if at least 50% of the residues belonging to the motif true positive matches are in disordered regions.

# 3) Statistics on motifs occurring in loop regions

Table 5a - Number of motifs predicted to be in loop regions accordingly to PSIPRED in the 25% non-redundant sequence dataset

| **PSIPRED** | **All** | **Significant SLiMs^(b)^** | **Known SLiMs^(c)^** | **Novel SLiMs^(c)^** |
| --- | --- | --- | --- | --- |
| H.sapiens | 902/1786 | 45/86 | 39/74 | 6/12 |
| *M.musculus* | 927/1812 | 63/98 | 56/82 | 7/16 |
| *R.norvegicus* | 904/1735 | 35/52 | 30/42 | 5/10 |
| *D.melanogaster* | 521/1241 | 14/26 | 11/22 | 3/4 |
| *C.elegans* | 491/881 | 31/35 | 28/32 | 3/3 |
| *E.coli* | 436/906 | 6/15 | 5/14 | 1/1 |
| *S.cerevisiae* | 438/800 | 14/19 | 11/14 | 3/5 |

A motif is assigned to a loop region if at least 50% of the residues belonging to the motif true positive matches are in loop regions.

**Table 5b** **- Number of motifs predicted to be in loop regions accordingly to PSIPRED in the 40% non-redundant sequence dataset.**

| **PSIPRED** | **All** | **Significant SLiMs^(b)^** | **Known SLiMs^(c)^** | **Novel SLiMs^(c)^** |
| --- | --- | --- | --- | --- |
| H.sapiens | 1102/2097 | 67/104 | 60/82 | 7/22 |
| *M.musculus* | 1061/2094 | 72/127 | 64/99 | 8/28 |
| *R.norvegicus* | 957/1863 | 38/72 | 31/57 | 7/15 |
| *D.melanogaster* | 602/1391 | 16/35 | 14/31 | 2/4 |
| *C.elegans* | 602/1050 | 25/32 | 22/26 | 3/6 |
| *E.coli* | 454/933 | 2/11 | 1/9 | 1/2 |
| *S.cerevisiae* | 493/889 | 13/20 | 13/17 | 0/3 |

A motif is assigned to a loop region if at least 50% of the residues belonging to the motif true positive matches are in loop regions.
