# Supplementary figures and images for: Exploiting Publicly Available Biological and Biochemical Information for the Discovery of Novel Short Linear Motifs
Source: PLoS One. 2011 Jul 20;6(7):e22270. doi: 10.1371/journal.pone.0022270 (PMC3140502; doi:10.1371/journal.pone.0022270)

Figure S1:  
(a)

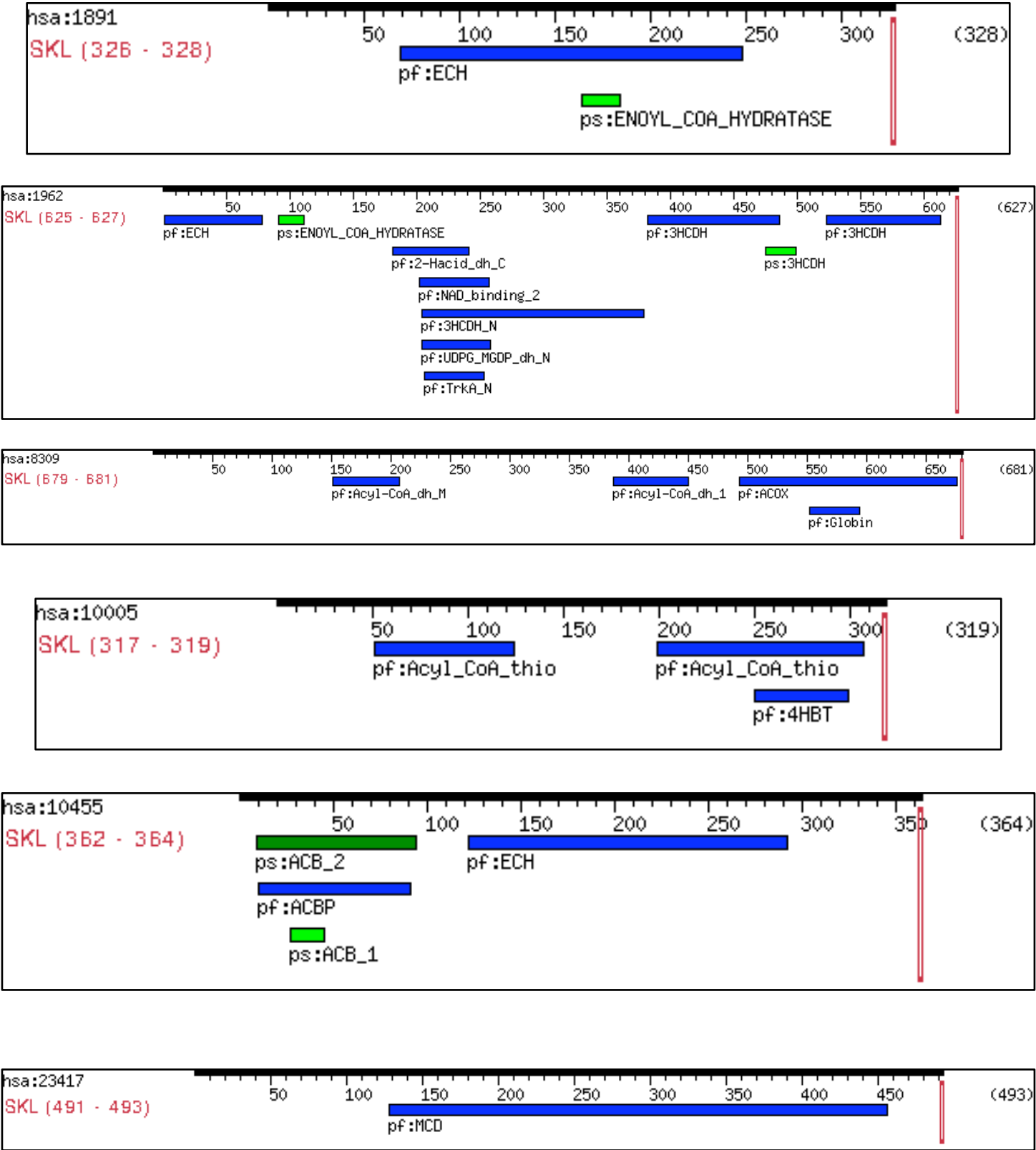

(b)

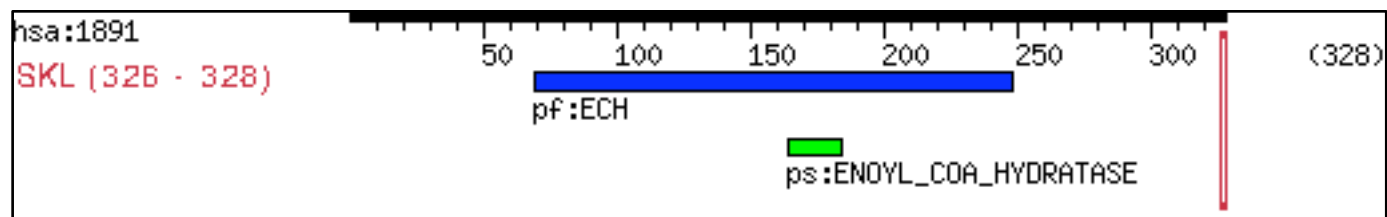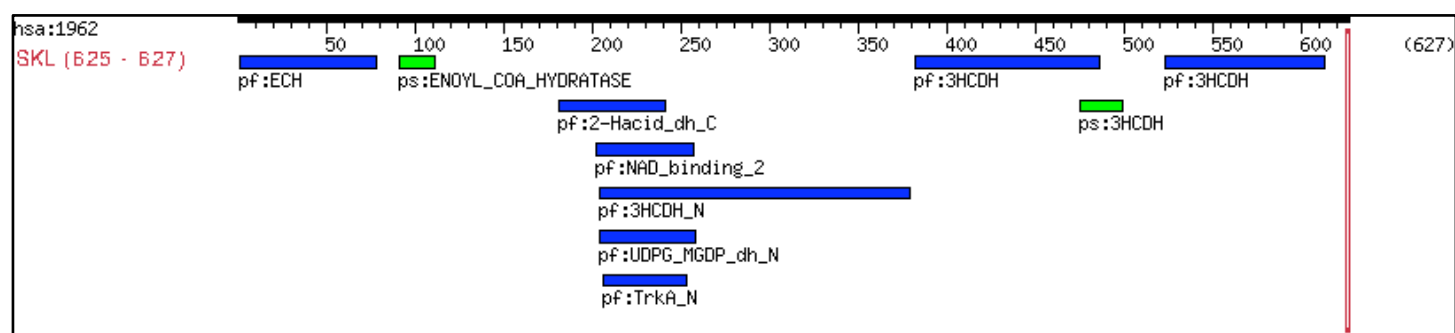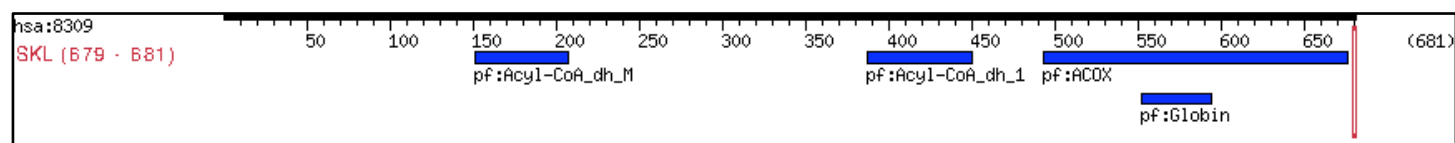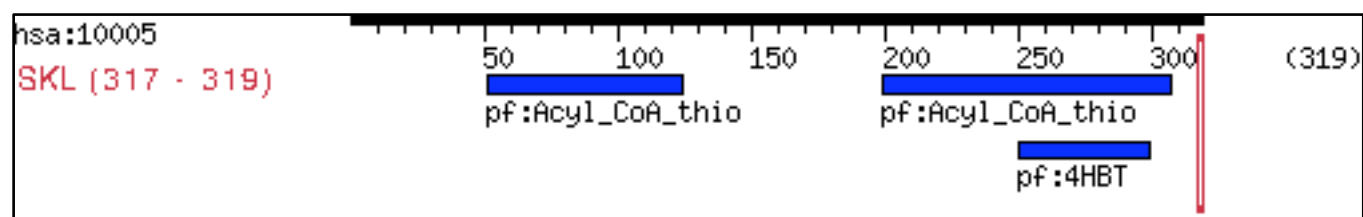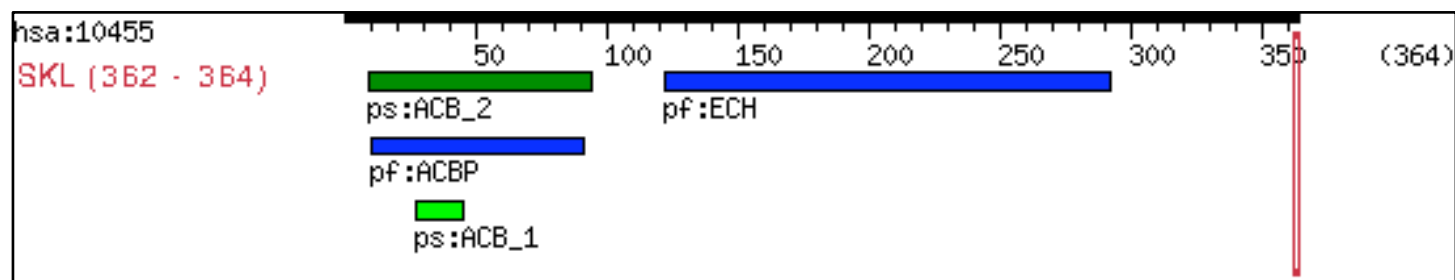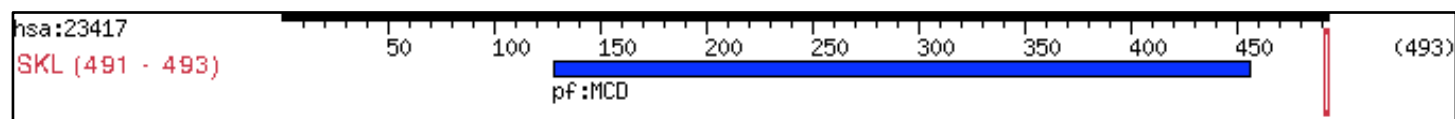

Supplement: Figure S1 — PROSITE and Pfam domain composition of hsa04146 KEGG pathway sequences matching the SKL$ motif. PROSITE and Pfam domain composition in the (a) 25% and (b) 40% non-redundant sequences belonging to the hsa04146 KEGG pathway and matching the SKL$ motif. Red bars indicate the position of the SKL$ motif in the sequence. Notice that there are no differences between (a) and (b). (PDF) [file pone.0022270.s006.pdf]

**Figure S2:**  
**(a)**

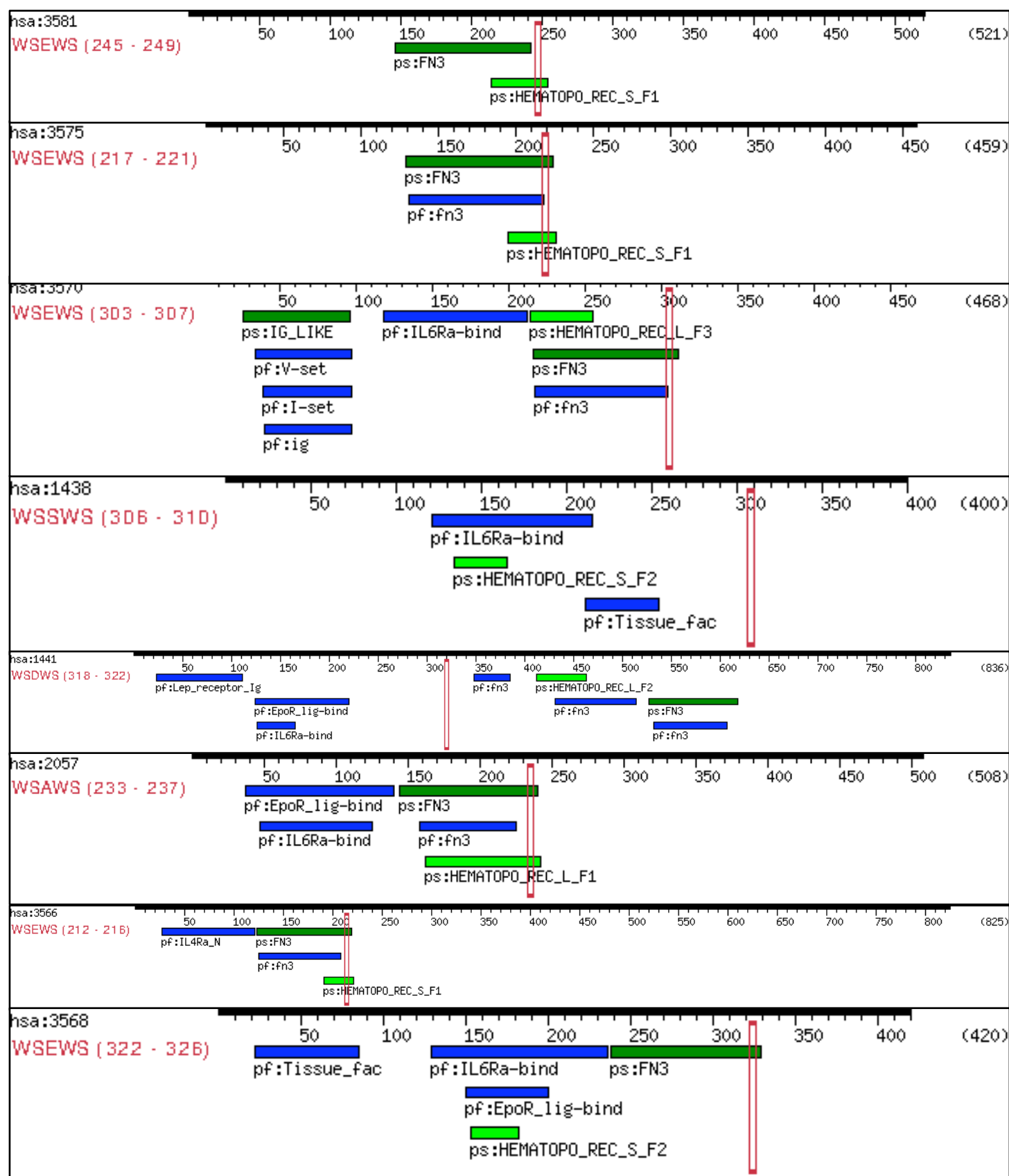

(b)

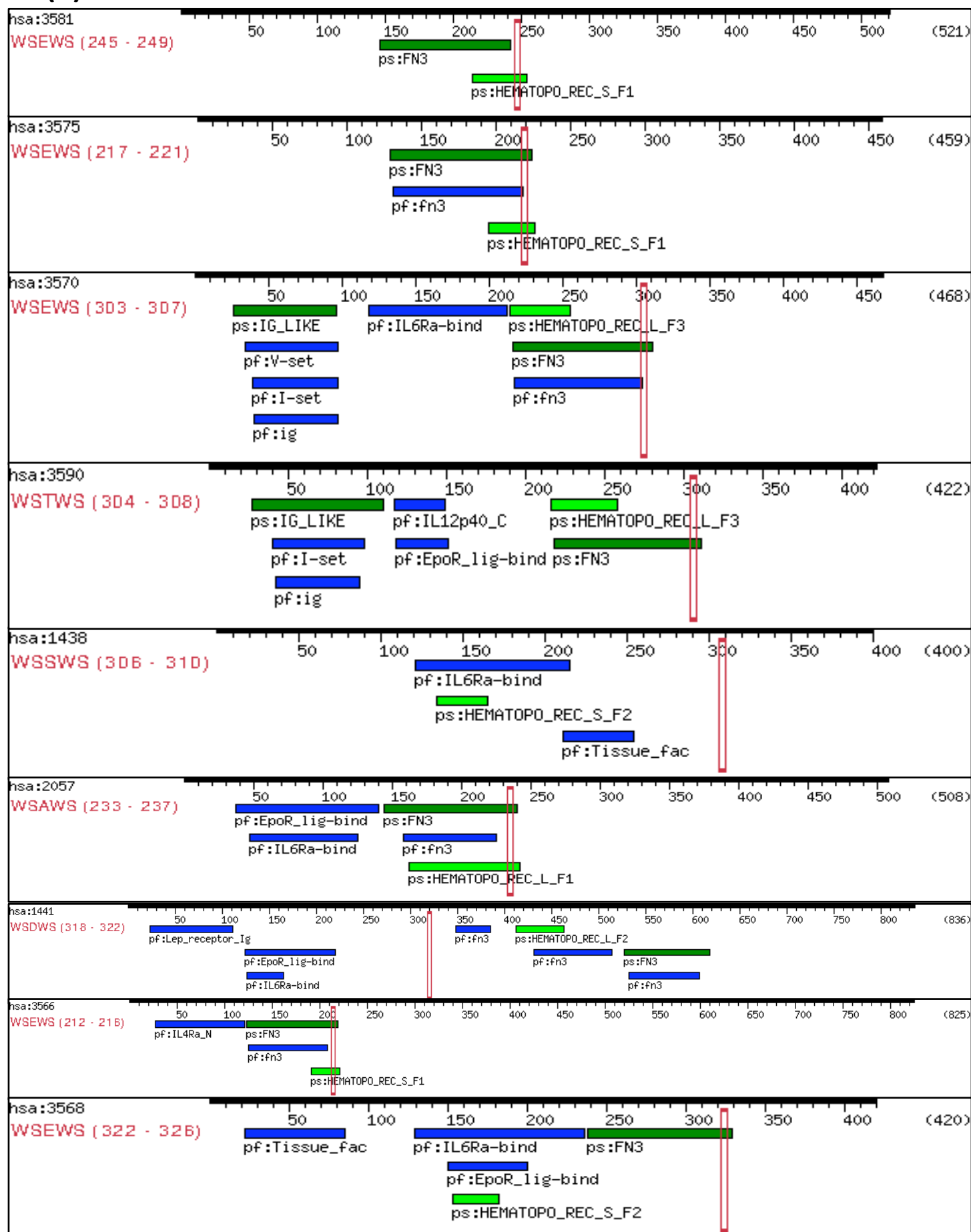

Supplement: Figure S2 — PROSITE and Pfam domain composition of hsa04640 KEGG pathway sequences matching the WS.WS motif. PROSITE and Pfam domain composition in (a) 25% and (b) 40% non-redundant sequences belonging to the hsa04640 KEGG pathway and matching the WS.WS motif. Red bars indicate the position of the WS.WS motif in the sequence. (PDF) [file pone.0022270.s007.pdf]
